# The BOADICEA model of genetic susceptibility to breast and ovarian cancers: updates and extensions

**DOI:** 10.1038/sj.bjc.6604305

**Published:** 2008-03-18

**Authors:** A C Antoniou, A P Cunningham, J Peto, D G Evans, F Lalloo, S A Narod, H A Risch, J E Eyfjord, J L Hopper, M C Southey, H Olsson, O Johannsson, A Borg, B Passini, P Radice, S Manoukian, D M Eccles, N Tang, E Olah, H Anton-Culver, E Warner, J Lubinski, J Gronwald, B Gorski, L Tryggvadottir, K Syrjakoski, O-P Kallioniemi, H Eerola, H Nevanlinna, P D P Pharoah, D F Easton

**Affiliations:** 1 Cancer Research UK, Genetic Epidemiology Unit, Department of Public Health and Primary Care, University of Cambridge, Cambridge, UK; 2 Section of Epidemiology, Institute of Cancer Research, Sutton, UK; 3 Section of Epidemiology, London School of Hygiene and Tropical Medicine, London, UK; 4 Academic Unit of Medical Genetics and Regional Genetics Service, St Mary's Hospital, Manchester, UK; 5 Centre for Research on Women's Health, University of Toronto, Toronto, Canada; 6 Department of Epidemiology and Public Health, Yale University School of Medicine, New Haven, CT, USA; 7 Faculty of Medicine, University of Iceland, Reykjavík, Iceland; 8 Icelandic Cancer Society, Reykjavík, Iceland; 9 Centre for Molecular, Environmental, Genetic and Analytic Epidemiology, The University of Melbourne, Melbourne, Victoria, Australia; 10 Genetic Epidemiology Laboratory, Department of Pathology, The University of Melbourne, Melbourne, Victoria, Australia; 11 Department of Oncology, Lund University Hospital, Lund, Sweden; 12 Fondazione IRCCS Istituto Nazionale Tumori, Milan, Italy; 13 IFOM Fondazione Istituto FIRC di Oncologia Molecolare, Milan, Italy; 14 Wessex Clinical Genetics Service, Princess Anne Hospital, Southampton, UK; 15 Department of Chemical Pathology, The Chinese University of Hong Kong, Hong Kong, People's Republic of China; 16 National Institute of Oncology, Budapest, Hungary; 17 Epidemiology Division, Department of Medicine, University of California – Irvine, Irvine, CA, USA; 18 International Hereditary Cancer Centre, Department of Genetics and Pathology, Pomeranian Academy of Medicine, Szczecin, Poland; 19 Laboratory of Cancer Genetics, Institute of Medical Technology, Tampere University Hospital, Tampere, Finland; 20 Department of Obstetrics and Gynecology, Helsinki University Central Hospital, Helsinki, Finland; 21 Cancer Research UK, Human Cancer Genetics Group, Department of Oncology, University of Cambridge, Cambridge, UK

**Keywords:** *BRCA1*, *BRCA2*, cancer risk model, genetic testing

## Abstract

Multiple genetic loci confer susceptibility to breast and ovarian cancers. We have previously developed a model (BOADICEA) under which susceptibility to breast cancer is explained by mutations in *BRCA1* and *BRCA2*, as well as by the joint multiplicative effects of many genes (polygenic component). We have now updated BOADICEA using additional family data from two UK population-based studies of breast cancer and family data from *BRCA1* and *BRCA2* carriers identified by 22 population-based studies of breast or ovarian cancer. The combined data set includes 2785 families (301 *BRCA1* positive and 236 *BRCA2* positive). Incidences were smoothed using locally weighted regression techniques to avoid large variations between adjacent intervals. A birth cohort effect on the cancer risks was implemented, whereby each individual was assumed to develop cancer according to calendar period-specific incidences. The fitted model predicts that the average breast cancer risks in carriers increase in more recent birth cohorts. For example, the average cumulative breast cancer risk to age 70 years among *BRCA1* carriers is 50% for women born in 1920–1929 and 58% among women born after 1950. The model was further extended to take into account the risks of male breast, prostate and pancreatic cancer, and to allow for the risk of multiple cancers. BOADICEA can be used to predict carrier probabilities and cancer risks to individuals with any family history, and has been implemented in a user-friendly Web-based program (http://www.srl.cam.ac.uk/genepi/boadicea/boadicea_home.html).

The risk of breast cancer in first-degree relatives of women with breast cancer is approximately two times higher than in women from the general population (Collaborative Group in Hormonal Factors in Breast Cancer, 2001). Mutations in the high-risk breast cancer-susceptibility genes *BRCA1* and *BRCA2* account for approximately 15% of this excess familial risk (Easton, 1999; Peto *et al*, 1999; Anglian Breast Cancer Study Group, 2000; Dite *et al*, 2003). We had previously derived a breast cancer susceptibility model, the Breast and Ovarian Analysis of Disease Incidence and Carrier Estimation Algorithm (BOADICEA), based on segregation analysis of breast and ovarian cancer occurrence in a combined data set, including a population-based series of 1484 breast cancer cases and 156 multiple case families from the United Kingdom (Antoniou *et al*, 2002, 2004). According to this model, genetic susceptibility to breast cancer is explained by the effects of *BRCA1* and *BRCA2* mutations, and the residual familial clustering is explained by the joint multiplicative effect of a large number of genes each of small effect (i.e., by a polygenic component). Direct evidence for the polygenic basis of the residual familial clustering not due to *BRCA1* and *BRCA2* mutations has more recently been provided by the identification of further loci that confer moderate risks, including mutations in *CHEK2*, *ATM*, *PALB2*, *BRIP1* (The CHEK2 Breast Cancer Case–Control Consortium, 2004; Renwick *et al*, 2006; Seal *et al*, 2006; Rahman *et al*, 2007) and the low-risk variants identified through genome-wide or candidate gene association studies (Cox *et al*, 2007; Easton *et al*, 2007; Hunter *et al*, 2007; Stacey *et al*, 2007).

The BOADICEA model can be used to estimate the likelihood of carrying a *BRCA1* or a *BRCA2* mutation, and the risks of developing breast or ovarian cancer. However, there are a number of limitations associated with the first version of BOADICEA. The model assumed that a fixed set of calendar period incidences applied to all cohorts, when breast cancer incidences have been increasing over time (Office for National Statistics, 2001; Tryggvadottir *et al*, 2006). Moreover, the incidences were assumed to change in 5-year intervals when in reality they change smoothly with age. As part of the model-fitting process, we also estimated the *BRCA1* and *BRCA2* breast and ovarian cancer risks, but these were based on a relatively small number of mutation-carrying families and were therefore imprecise (Antoniou *et al*, 2002, 2004).

Finally, the model took into account only the occurrence of a first breast or a first ovarian cancer and the risks of second or subsequent cancers were ignored. *BRCA1* and *BRCA2* mutations are associated with increased risk of cancer at several sites other than female breast and ovary. The strongest evidence is for prostate and pancreatic cancer in *BRCA2* carriers, which has been consistently found in multiple studies (The Breast Cancer Linkage Consortium, 1999; Tulinius *et al*, 2002; Edwards *et al*, 2003; Kirchhoff *et al*, 2004; van Asperen *et al*, 2005; Risch *et al*, 2006; Tryggvadottir *et al*, 2007). There is also more limited evidence for an increased risk of cancer of the gall bladder, bile duct, stomach and malignant melanoma (The Breast Cancer Linkage Consortium, 1999; Edwards *et al*, 2003; Kirchhoff *et al*, 2004; van Asperen *et al*, 2005; Risch *et al*, 2006). *BRCA1* carriers have been found to have elevated risks of colorectal, pancreatic, prostate, testicular and uterine cancer (Ford *et al*, 1994; Brose *et al*, 2002; Thompson and Easton, 2002; Risch *et al*, 2006). The colorectal cancer risk, however, has not been consistently replicated, while the prostate and pancreatic cancer risks are lower than in *BRCA2* carriers. In addition, male *BRCA1* and (to a greater extent) *BRCA2* carriers are at an increased risk of developing breast cancer (Thorlacius *et al*, 1997; Thompson and Easton, 2001; Risch *et al*, 2006; Tai *et al*, 2007), (DF Easton, unpublished data). Incorporation of these additional cancer phenotypes into the model should provide greater discrimination between *BRCA1* and *BRCA2* mutation carriers and noncarriers.

In this paper, we have attempted to improve BOADICEA by analysing additional population-based data and by extending the model to account for the risk of cancer after the first diagnosis and the risks of cancer at sites other than breast and ovary.

## MATERIALS AND METHODS

We combined the data from the two studies used in the initial model development with data from three additional published population-based studies. Specifically, the present analysis includes the following data sets:

*(a) The Anglian Breast Cancer Study (ABC, now SEARCH).* The families were identified through 1484 women with breast cancer diagnosed before the age of 55 years and registered in the East Anglian Cancer Registry between 1991 and 1996. These index cases were invited to provide blood samples and complete an epidemiological questionnaire, including family history of cancer in all first-degree relatives. The blood samples were tested for germline mutations in *BRCA1* and *BRCA2* using conformation-sensitive gel electrophoresis (CSGE). Mutations were confirmed by sequencing. These data were used in the initial model development and the study is described in more detail elsewhere (Anglian Breast Cancer Study Group, 2000; Antoniou *et al*, 2001).

*(b) UK National Case–Control Study (UK).* Women with breast cancer were identified through two UK population-based case–control studies. The first study involved 755 patients diagnosed under the age of 36 years and registered between 1982 and 1985. The second study included 644 patients diagnosed from age 36 to 45 years and registered between 1988 and 1989. These index cases provided family history information of breast and ovarian cancer and were later contacted to provide blood samples. DNA was screened for germline mutations in *BRCA1* and *BRCA2* by heteroduplex analysis. Again, mutations were confirmed by sequencing. In all, 617 samples were tested for *BRCA1* and *BRCA2* mutations and were included in our analysis. This data set is described in detail elsewhere (Peto *et al*, 1999).

*(c) The Manchester Study.* Women diagnosed with breast cancer at or before the age of 30 years were recruited via the North West Regional Cancer Registry (UK) between 1980 and 1997. A total of 99 index cases provided blood samples, which were screened for mutations in *BRCA1* and *BRCA2* using a combination of the Protein truncation test, single-strand conformation polymorphism/heteroduplex analysis and fluorescent chemical cleavage of mismatch analysis. Three-generational pedigrees were constructed through interviews and were augmented with data from hospital notes. The study is described in Lalloo *et al* (2003).

*(d) Multiple case families: ‘British’ (B) families.* In all, 156 families were ascertained in response to national publicity in the United Kingdom and by referral by oncologists or general practitioners. Eligibility was restricted to families with at least two breast cancer cases, one or more diagnosed before the age of 50 years. Occurrence of cancer and follow-up was recorded on all family members. One or more individuals from each family provided blood samples, which were analysed for *BRCA1* and *BRCA2* mutations using CSGE (Antoniou *et al*, 2002). This set of families was also used in the initial development of BOADICEA.

*(e) Meta-analysis families (BRCA families).* This data set included pedigree data from *BRCA1* and *BRCA2* mutation carriers identified in 22 population-based studies of breast or ovarian cancer patients reported by Antoniou *et al* (2003). A study was eligible for the meta-analysis if it was based on mutation testing of a series of index cases diagnosed with either breast (male or female) or epithelial ovarian cancer and who were unselected for family history of cancer. In each study, the index cases had to be tested for *BRCA1* and/or *BRCA2* mutations by systematic screening, and family history information had to be available on all first-degree relatives of identified mutation carriers. To avoid replication, the families of mutation carriers identified through the ABC, UK and Manchester studies were not considered to be part of the BRCA families for the present analysis. A total of 429 families of *BRCA1* and *BRCA2* mutation carriers were included in the present analysis.

*BRCA1* and *BRCA2* mutations were considered to be disease causing if they were classified pathogenic according to the generally accepted criteria (http://research.nhgri.nih.gov/projects/bic/). For consistency across the population-based studies (ABC, United Kingdom, Manchester and BRCA), family history information was restricted to the first-degree relatives of the index cases.

### Segregation analysis

Model fitting was performed using complex segregation analysis of breast and ovarian cancer occurrences in the combined set of families described above. Individuals were followed from birth and were censored at the age of cancer occurrence, age at death or at the age of 70 years whichever occurred first. Female patients with no age information or no year of birth were censored at age 0 (692 female patients in the combined data set).

In the initial development of BOADICEA, a number of different genetic models were investigated for the genetic susceptibility to breast cancer (Antoniou *et al*, 2002). It was found that the most parsimonious model was one that incorporated the simultaneous effects of *BRCA1*, *BRCA2* and a polygenic component representing the combined multiplicative effect of multiple loci of small effect. This model is consistent with the recent discovery of multiple low-risk susceptibility genes (and the failure to identify any further ‘high-risk’ loci by linkage) (Smith *et al*, 2006; Easton *et al*, 2007; Hunter *et al*, 2007; Stacey *et al*, 2007). Although it is likely that the genetic causes of breast cancer are more complicated, for the present analysis we focused only on polygenic models for the residual familial clustering of breast cancer other than that due to mutations in *BRCA1* and *BRCA2*.

The breast cancer incidence for individual *i* at age *t* was assumed to be birth cohort specific, and to depend on the underlying *BRCA1* or *BRCA2* genotype and polygenotype through a model of the form *λ*_*i*_(*t*)=*λ*_0_(*t*)exp(*G*_*i*_(*t*)+*P*_*i*_(*t*)), where *λ*_0_(*t*) is the baseline incidence for the cohort, *G*_*i*_(*t*) represents the major gene effect at age *t* (*BRCA1* carrier, *BRCA2* carrier or noncarrier) and *P*_*i*_(*t*) is the polygenic effect assumed to be normally distributed with mean zero and variance *σ*^2^(*t*). The polygenic component was approximated by the hypergeometric polygenic model (Antoniou *et al*, 2001; Lange, 2002). More details about the implementation of this approximation in MENDEL can be found in Antoniou *et al* (2001). Under the above model, the polygenotype is assumed to modify the disease risk in *BRCA1* and *BRCA2* carriers. In this context, that is, when *P*_*i*_(*t*) acts on *BRCA1* and *BRCA2* background, the polygenic component is referred to as the ‘modifying’ component. In this analysis, we generalised the model to allow for different polygenic and modifying variances in mutation carriers and noncarriers. We also fitted models in which the polygenic and modifying variance was age dependent.

### Calendar period- and cohort-specific incidences

The breast and ovarian cancer incidences were assumed to be calendar period and cohort specific, based on the incidences for England and Wales (Cancer in five continents volumes I–VIII, Doll *et al*, 1966, 1970; Waterhouse *et al*, 1976, 1982; Muir *et al*, 1987; Parkin *et al*, 1992, 2002). Five birth cohorts were assumed for this purpose (<1920, 1920–1929, 1930–1939, 1940–1949, and 1950 or after), and the incidences were derived by assuming that the female patient was born at the midpoint of the relevant birth cohort (1915 for the first cohort and 1955 for the last cohort). The overall incidences were constrained to agree with the population incidences for each cohort separately (Antoniou *et al*, 2001). The rate ratios associated with the major gene and polygenic effects (*G*_*i*_(*t*), *P*_*i*_(*t*)) were assumed not to vary by birth cohort. Owing to this constraint, the estimated incidences for *BRCA1* and *BRCA2* carriers and noncarriers were themselves cohort specific.

### Incidence smoothing

Published incidences are reported in 5-year intervals, which can result in large variations in the incidences between adjacent age intervals. This is particularly an issue for *BRCA1* and *BRCA2* carriers for whom incidence increases rapidly with age. Since it is more plausible to assume that incidences vary continuously with age, we smoothed the population incidences using locally weighted regression techniques (Royston, 1991). This method was chosen because it follows the ‘locality’ of the data as opposed to polynomial smoothing, which is a global graduation technique, is influenced by the extreme points (young and old ages) and is not as flexible. Smoothing was carried out using the statistical software Stata (Stata Corporation, College Station, Texas, USA). The method involves running a regression at each age *t*_*i*_, using the data for age *t*_*i*_ and a small amount of data near *t*_*i*_. The proportion of data (bandwidth) used in the regression specifies the degree of smoothness. Various degrees of smoothness were investigated and the resulting set of incidences was compared with the original set of incidences for adherence. As a smoothness criterion, we used the sum of the absolute values of the third-order finite differences of the smoothed incidences: 
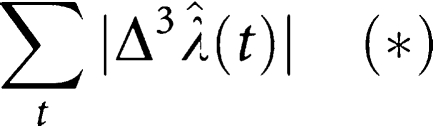
 where 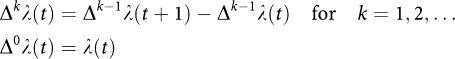


Smaller values of the sum (*) correspond to smoother incidence curves. Consistency of the model with the data was assessed using a *χ*^2^ test statistic, treating the smoothed incidences as expected values. However, formal tests of significance were not performed because of the difficulty in determining the correct number of degrees of freedom for the test.

### *BRCA1* and *BRCA2* relative risks

Two types of models were assumed for the *BRCA1* and *BRCA2* log-relative risks (*G*_*i*_(*t*)) for both breast and ovarian cancer. Our primary analysis involved fitting models in which the relative risks are assumed to be constant within each decade of age (20–29, 30–39, 40–49, 50–59 and 60–69 years). Once the most parsimonious model for the form of the polygenic and modifying variance was chosen, we fitted additional models in which the log-relative hazards were piecewise linear functions of age (see Appendix), so that the resulting *BRCA1* and *BRCA2* incidences were continuous functions of age.

### Adjustment for ascertainment

For the ABC, UK and Manchester families, we adjusted for ascertainment by maximising the conditional likelihood of observing the phenotypes and genotypes in the families, given the disease status and age at diagnosis of the index case. For the *BRCA1*- and *BRCA2*-positive families, we maximised the conditional likelihood of observing the disease phenotypes and genotypes in the family, given the disease status, age at diagnosis and mutation status of the index case. Since the ‘B’ families were identified through multiple-affected individuals, we maximised the likelihood of all phenotypes and genotypes in the family conditional on all the phenotypic information for the family.

### Sensitivity of the mutation testing

To allow for the fact that not all mutations could be detected by the screening methods used, we allowed in our analysis for a sensitivity of mutation testing parameter, giving the probability of detecting a mutation if one exists. We assumed that 70 and 80%, respectively, of the disease-causing mutations in *BRCA1* and *BRCA2* could be detected by the methods used. The sensitivity parameter only applied for the first screened individual from each family (the index cases). Variants of uncertain significance (VUSs) were assumed to be equivalent to a *BRCA1*- and *BRCA2*-negative test, since this is how they are treated in clinical genetics and in analyses testing the goodness of fit of the model. Although some such variants may be pathogenic, this effect is allowed for in the model by the mutation sensitivity parameter (i.e., the incomplete sensitivity is partly due to pathogenic mutations classified as VUSs). For the relatives of index patients who were screened for family-specific mutations, we assumed that the test was 100% sensitive.

### Model comparisons

Nested models were compared against each other using the likelihood ratio test. The Akaike Information Criterion (AIC) was used to compare non-nested models (Akaike, 1974).

### Parameter estimation

The models were parameterised in terms of the polygenic and modifying variances, the *BRCA1* and *BRCA2* allele frequencies in the population and the natural logarithm of the ratios of the breast and ovarian cancer incidences in *BRCA1* and *BRCA2* mutation carriers to the population incidences (relative hazards). Parameters were estimated by maximum likelihood, and their variances were obtained from the observed information matrix. To obtain confidence intervals for parameters with restricted ranges (e.g., allele frequencies) we used transformations to obtain parameters that are likely to be more normally distributed (Antoniou *et al*, 2001).

## RESULTS

Table 1 summarises the total number of families by the mutation status of the index case. In all, 2785 families were included in the analysis, of which 301 were *BRCA1* positive and 236 were *BRCA2* positive. Table 2 shows the number of breast and ovarian cancer patients by age at diagnosis for the relatives of the index cases.

The smoothed incidences used in the segregation analyses were based on a bandwidth of 0.2. This value provided a compromise between smoothness and adherence to the original published incidences (data not shown). The graduated calendar- and cohort-specific incidences included the age-specific features for all cohorts observed in the general population (data not shown) (Office for National Statistics, 2001). On the basis of the graduated incidences, the risk of breast cancer in the general population by the age of 80 years is 8.9, 9.8, 10.4, 10.9 and 11.0% for female patients born prior to 1920, between 1920–1929, 1930–1939, 1940–1949, and 1950 and after, respectively.

We first fitted three models, with different assumptions about the polygenic and modifying variances (Table 3). For these models, the *BRCA1* and *BRCA2* relative hazards were assumed to be constant within each 10-year interval: 20–29, 30–39, 40–49, 50–59 and 60–69 years. In the first model, the polygenic and modifying variances were constrained to be equal. In the second model, the polygenic variance was allowed to be different from the modifying variance, but the latter was constrained to be the same for *BRCA1* and *BRCA2* carriers. In the third model, the polygenic variance and the *BRCA1* and *BRCA2* modifying variances were all allowed to vary. In each of these models, the polygenic and modifying variance were assumed to be constant with age. We found no evidence that the *BRCA1*- and *BRCA2*-modifying variances were different from each other (*P*=0.76). The modifying variance was estimated to be somewhat lower than the polygenic variance (1.55 *vs* 2.02), but the difference was not significant (*P*=0.63). When a single polygenic/modifying variance was assumed, it was estimated to be 1.99 (95% CI: 1.54–2.57). This model had the lowest AIC value of the three models (7948.284). The *BRCA1* and *BRCA2* parameter estimates for this model are given in Table 4.

We then fitted three further models, for which the polygenic variance was allowed to vary by age group (20–29, 30–39, 40–49, 50–59 and 60–69 years; Table 3). When the modifying variance was restricted to be equal to the polygenic variance, this model did not fit significantly better than the model with the same constant polygenic and modifying variance (*P*=0.30). The other two models assumed separate constant modifying variances, which were either constrained to be equal or different among *BRCA1* and *BRCA2* mutation carriers. These models also did not improve the fit significantly, compared with the model with a constant polygenic/modifying variance (*P*=0.53 and 0.65). The latter two models assumed constant modifying variances because models with varying modifying variances resulted in unbounded estimates.

Despite the lack of a significant improvement in fit, the parameter estimates suggest that the polygenic variance may decrease with age. We explicitly allowed for this hypothesis by fitting a model in which the polygenic and modifying variances were the same linear function of age, that is, *σ*_*p*_^2^(*t*)=*σ*_*m*_^2^(*t*)=*α*+*βt*, where *t* represents the age in years, and estimated the parameters *α* (=4.86, 95% CI: 1.8–7.9) and *β* (=−0.06, 95% CI: −0.12 to −0.0002). Compared with the model with a constant variance, there was some marginal evidence that this model fitted better (*P*=0.049).

To investigate further the properties of these models, we computed the age-specific familial relative risks (FRRs) for an individual with an affected mother predicted by these two models as described elsewhere (Antoniou *et al*, 2004). We then contrasted these against the observed FRR estimated by epidemiological studies (Collaborative Group in Hormonal Factors in Breast Cancer, 2001). The predicted FRRs were closer to the observed values for the model with a polygenic/modifying variance that decreased with age than the model with a constant variance (Table 5). When a constant polygenic variance was assumed, the predicted FRRs decreased markedly at young ages in line with the observed values, but were still high at ages 55 years and older. The model with a linearly decreasing polygenic variance predicted an FRR, which was close to the observed values at all ages.

### *BRCA1* and *BRCA2* incidence smoothing

**Figure 1:**
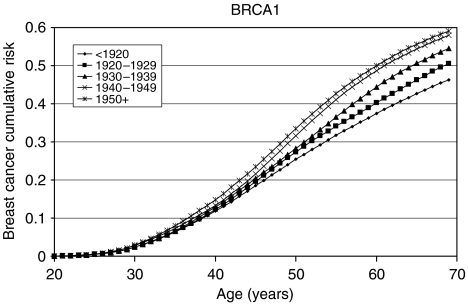
Average cumulative breast cancer risks for *BRCA1* mutation carriers by birth cohort assumed in BOADICEA.

**Figure 2:**
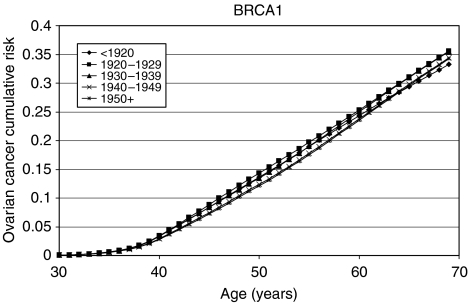
Average cumulative ovarian cancer risks for *BRCA1* mutation carriers by birth cohort assumed in BOADICEA.

**Figure 3:**
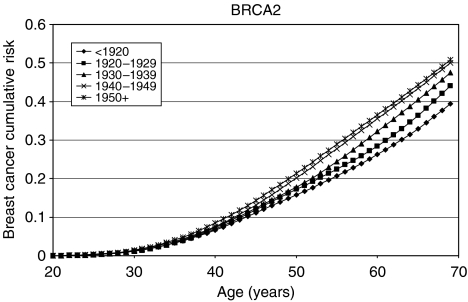
Average cumulative breast cancer risks for *BRCA2* mutation carriers by birth cohort assumed in BOADICEA.

**Figure 4:**
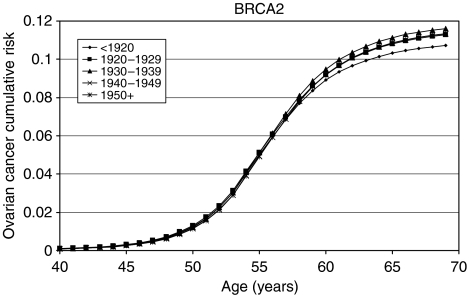
Average cumulative ovarian cancer risks for *BRCA2* mutation carriers by birth cohort assumed in BOADICEA.

The model with a linearly decreasing polygenic/modifying variance was extended to allow for the *BRCA1* and *BRCA2* log-relative hazards to be piecewise linear functions of age (see Materials and Methods, Appendix and Table 6). The allele frequencies of *BRCA1* and *BRCA2* mutations in the general population were estimated to be 0.06% (95% CI: 0.04–0.10%) and 0.10% (95% CI: 0.07–0.16%), respectively. These correspond to population carrier frequencies of 0.12% for *BRCA1* and 0.20% for *BRCA2*. The average cumulative risks of breast and ovarian cancer in *BRCA1* and *BRCA2* mutation carriers based on this model are shown in Figures 1, 2, 3 and 4. The average cumulative risk of breast cancer in *BRCA1* carriers over all possible modifiers was estimated to be 46% by the age of 70 years for women born before 1920, rising to 59% for women born after 1950. On the basis of the 5th and 95th percentiles of the distribution of the polygenic/modifying component, the estimated cumulative breast cancer risks were 7.2 and 98%, respectively, for carriers born before 1920, rising to 12.0 and 99.9% for carriers born after 1950. The average cumulative risks of breast cancer in *BRCA2* mutation carriers by the age of 70 years were estimated to be lower, 39% for women born before 1920 (6.3 and 93% at the 5th and 95th percentiles, respectively) rising to 51% for those born after 1950 (9.8 and 98.7% at the 5th and 95th percentiles, respectively). The estimated ovarian cancer risks were highest for women born between 1930 and 1939, but the variation across the birth cohorts was smaller than for breast cancer. For *BRCA1* mutation carriers, the ovarian cancer risk by the age of 70 years was estimated to be 33% for women born prior to 1920, rising to 36% for those born between 1920 and 1939, and then dropping to 34%. For *BRCA2* mutation carriers, the corresponding risks were estimated to be 11, 12 and 11%, respectively. The predicted age-specific FRRs under this model are shown in Table 5. These are generally similar to those generated using piecewise constant hazard ratios, but slightly closer to the observed values.

### Risks of cancers at other sites and risks of second cancers

Since reliable data on these additional cancer types were not available in the main data set used to derive BOADICEA, we used instead estimated risks derived from the largest published studies (see Table 7). We incorporated only prostate and pancreatic cancer, and male breast cancer, for which the evidence for association and estimates of risk were most reliable. We assumed that conditional on the genotype, the age-specific probability of developing a particular cancer was independent of the probability of developing any other type of cancer. The incidences of prostate and pancreatic cancer for *BRCA1* and *BRCA2* mutation carriers were obtained by multiplying the cohort and calendar period age-specific incidence rates from the general population. Noncarriers were assumed to develop these cancers according to the population incidences.

The risk of breast cancer in female first-degree relatives of male breast cancer patients has been estimated to be 2.4 times greater than the risk in the general population (Basham *et al*, 2002), consistent with a common genetic susceptibility to male and female breast cancer. However, *BRCA1* and *BRCA2* alone cannot explain all the observed FRRs (Basham *et al*, 2002). To allow for this residual effect, we assumed that the polygenic component in the model also applied to male patients. The male breast cancer polygenic variance was chosen such that the predicted FRR to daughters of male breast cancer patients was equal to 2.4. In choosing this, the overall male breast cancer incidences over the *BRCA1*, *BRCA2* (Table 7) and polygenic effects were constrained to agree with the population incidences. On the basis of this approach, the male breast cancer polygenic variance was chosen to be 1.96, close to that estimated for female patients at young ages.

**Figure 5:**
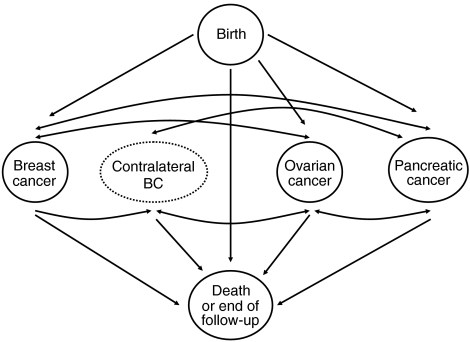
The extended BOADICEA transition model for female patients.

We have also extended the model to allow for the risks of other cancers after the first cancer diagnosis, including the risk of contralateral breast cancer. We assumed that the increased risk of contralateral breast cancer, or of any other cancer after the first diagnosis (relative to the population rates), was entirely due to the susceptibility as defined by the model (i.e., no additional variation in risk). On the basis of this assumption, the contralateral breast cancer incidence after the first breast cancer, given the genotype, is half the breast incidence assumed in the standard model (since only one breast is at risk). Similarly, the incidence of a cancer at another site, after a first cancer diagnosis, was assumed to be the same as if the preceding cancer had not occurred, consistent with the assumption that the site-specific cancer risks are independent conditional on the genotype. The transition model for female patients in this extended BOADICEA model is depicted in Figure 5. A similar model applies to male patients (including prostate cancer but excluding ovarian cancer).

## DISCUSSION

In this report, we have updated and extended our previously published model BOADICEA using additional data. There are several additional features in the updated model. The breast and ovarian cancer incidences in *BRCA1* and *BRCA2* mutation carriers are now based on a much larger number of mutation-carrying families and are, therefore, more reliable; the variance of the polygenic component is now age dependent as opposed to constant; and the incidences vary gradually with age and are cohort and calendar period specific. In addition, the model has been extended to allow for the risks of male breast, prostate and pancreatic cancer, and the risks of other cancers after a first diagnosis.

We have shown that the updated version, with a polygenic variance that decreases linearly with age, predicts accurately the FRR of breast cancer observed in epidemiological studies (Collaborative Group in Hormonal Factors in Breast Cancer, 2001). Such a model is consistent with the hypothesis that a large number of variants increase the rate at which key mutational events occur, and the increased relative risk conferred by at least some variants is higher at younger ages. Direct evidence for a polygenic component is provided by the recent identification of at least seven common variants through genome-wide association and candidate gene studies (Cox *et al*, 2007; Easton *et al*, 2007; Hunter *et al*, 2007; Stacey *et al*, 2007). For the two strongest associations identified by Easton *et al* (2007) (*FGFR2* and *TNRC9*), the per allele odds ratio was higher below the age of 40 years, although not significantly so. Other studies have found that the relative risks associated with *CHEK2* 1100delC and *ATM* mutations are somewhat higher at young ages (The CHEK2 Breast Cancer Case–Control Consortium, 2004; Thompson *et al*, 2005).

We investigated models that allowed for different polygenic (modifying) variances for *BRCA1* and *BRCA2* mutation carriers. The point estimate of the *BRCA1* variance was lower than of the polygenic variance for noncarriers, while that of the *BRCA2* variance was more similar to the latter. This would be consistent with recent findings that the established breast cancer susceptibility variants at the *FGFR2* and *MAP3K1* loci are associated with the risk of breast cancer in *BRCA2* but not *BRCA1* carriers (Antoniou *et al*, in press b). The latter observations may reflect differences in susceptibility by tumour characteristics, since some loci confer susceptibility only to ER-positive disease (Garcia-Closas, in press). However, the power to distinguish between these models, even with the current large data set, was limited, and we could not reject a model with equal polygenic and modifying variances. This would be consistent with the hypothesis that most ‘polygenes’ confer similar relative risks in *BRCA1* carriers, *BRCA2* carriers and noncarriers.

As part of the model fitting, we have also re-estimated the breast and ovarian cancer risks in *BRCA1* and *BRCA2* carriers. The majority of the current data came from families of unselected series of cases that had been previously used to estimate the *BRCA1* and *BRCA2* penetrance (Antoniou *et al*, 2003). The present analyses differed in the following three important respects: they were cohort and calendar period specific; they were based on incidences, which vary continuously with age; and, most importantly, they were estimated while allowing for other residual familial effects (i.e., polygenic-modifying component). Although a direct comparison with the breast cancer estimates in the original report is not strictly valid, the present breast cancer estimates are generally lower. There is a straightforward explanation for this difference. The present breast cancer risks (Figures 1, 2, 3 and 4) represent the risks averaged over all possible polygenic and modifying effects and would therefore be applicable to a randomly chosen *BRCA1* or *BRCA2* mutation carrier (i.e., for a carrier without any knowledge of her family history). In contrast, the estimates in Antoniou *et al* (2003) represent the breast cancer risks among *BRCA1* and *BRCA2* mutation carriers who have an affected first-degree relative (affected with breast cancer in most cases). Under the BOADICEA model, these women would be expected to have higher than average breast cancer risks, as demonstrated elsewhere (Antoniou *et al*, 2004) and as other researchers have pointed out (Begg, 2002). An important consequence of these arguments is that there is no single set of penetrance estimates that applies to all carriers. The range of breast cancer cumulative risks given by the distribution of the polygenic/modifying component implies that the cancer risks depend strongly on the genotypes at modifying loci – not just the presence of a mutation – and these can be much higher or much lower than the average estimates. To utilise the full range of these risk estimates would require the modifying genes to be identified, but, in the meantime, the results indicate that family history in addition to mutation status should be taken into account in genetic counselling. The BOADICEA model allows this level of sophistication. This feature is also consistent with the recent results of Begg *et al* (2008), who also demonstrated that breast cancer risks vary between families where a *BRCA1* or *BRCA2* mutation has been identified.

The updated version of BOADICEA was one of the models used in a recent validation study of *BRCA1* and *BRCA2* carrier prediction algorithms (Antoniou *et al*, in press a) using a large series of families seen in the UK genetics clinics. The BOADICEA model was compared against other models including the genetic risk models BRCAPRO (Parmigiani *et al*, 1998) and IBIS (Tyrer *et al*, 2004). It was found to be the most accurate model in terms of predicting the observed number of mutations in total, and across the whole range of probabilities of being a mutation carrier, and had the highest power to discriminate between mutation carriers and noncarriers. This, taken together with the observation that BOADICEA also predicts well the FRRs of breast cancer, provides confidence that BOADICEA is a well-validated and well-calibrated model that can be a useful tool for genetic counselling individuals with family history of breast cancer. However, further validation studies will be important to evaluate the ability of this (and other) models to predict the prospective risk of developing breast or ovarian cancer.

The current version of BOADICEA has now been implemented as a user-friendly Web-based program (http://www.srl.cam.ac.uk/genepi/boadicea/boadicea_home.html). Users can either create a pedigree online, or can upload a pedigree file. The program allows for families of any size or structure; pedigrees built online are restricted to first- and second-degree relatives but uploaded files can be of arbitrary complexity. It has been shown that relatives more distant than second degree can provide important information for risk models (Antoniou *et al*, 2005; Barcenas *et al*, 2006). The BOADICEA returns both predicted probabilities of carrying a *BRCA1* or a *BRCA2* mutation, and risks (by the age of up to 80 years) of developing breast or ovarian cancer for unaffected individuals, or the risk of contralateral breast cancer or ovarian cancer for those who have already developed a first breast cancer. The code has also been modified to allow for the possibility that the individual is of Ashkenazi Jewish origin. This case requires separate consideration owing to the high prevalence of three founder mutations in this population (Satagopan *et al*, 2001), so that estimates based on allele frequencies for the United Kingdom would be misleading. For this purpose, we used the *BRCA1* and *BRCA2* mutation prevalence for young controls (*BRCA1*: 1.6% and *BRCA2*: 1.2%) reported in Satagopan *et al* (2001). The cancer risks among *BRCA1* and *BRCA2* mutation carriers, and the polygenic variance, were assumed to be the same in the Ashkenazi and non-Ashkenazi versions. Similar modification will be required for other populations where the frequencies of *BRCA1* and *BRCA2* mutations are different (e.g., the Icelandic). These will be implemented at a later stage.

There are several directions in which our model can be extended, and these may lead to improvements in discriminatory power and more accurate predictions of cancer risk. Now that some of the loci that may comprise the ‘polygenic’ component have been identified, it will be possible to incorporate their specific effects into the model, allowing these additional loci to be used in counselling. Other extensions that remain challenges include the incorporation of variation in risk by mutation type; extensions to other populations with different risks and/or mutation frequencies; inclusion of data on pathological subtypes (for example, ‘basal’ breast cancer); tumour histological characteristics; and the inclusion of hormonal, reproductive and lifestyle risk factors.

## Figures and Tables

**Table 1:** Number of families included in the analysis by the mutation status of the index cases

**Study**	***BRCA1* carriers**	***BRCA2* carriers**	**Noncarriers**	**Total**
ABC	8	15	1461	1484
UK	16	14	587	617
Manchester	9	7	83	99
B families	21	18	117	156
BRCA	247	182	NA	429
				
Total	301	236	2248	2785

NA=not applicable.

**Table 2:** Number of breast and ovarian cancer cases among the relatives of index cases, by the age at diagnosis and the mutation status of the index case

	***BRCA1* carriers**		***BRCA2* carriers**		**Noncarriers**	
**Age group (years)**	**BC**	**OC**	**BC**	**OC**	**BC**	**OC**
<20	0	0	0	0	0	0
20–29	6	7	5	0	7	4
30–39	58	25	34	0	79	2
40–49	84	10	55	2	180	10
50–59	36	9	46	13	135	12
60–69	19	9	28	3	96	5
70+	12	3	20	4	78	12

BC=breast cancer; OC=ovarian cancer.

**Table 3:** Segregation analysis models fitted for the polygenic and modifying variances

**Model**	**Age (years)**	***σ_p_*^ 2^(*t*) (95% Cl)**	***σ_m_*_1_^ 2^(*t*) (95% Cl)**	***σ_m_*_2_^ 2^(*t*) (95% Cl)**	**Log-lik**
*σ*_*p*_ ^2^=*σ*_*m*_ ^2^	20–69	1.99 (1.5–2.6)	1.99 (1.5–2.6)	1.99 (1.5–2.6)	−3951.142
*σ*_*p*_ ^2^, *σ*_*m*_ ^2^	20–69	2.02 (1.6–2.6)	1.55 (0.6–4.4)	1.55 (0.6–4.4)	−3951.023
*σ*_*p*_ ^2^, *σ*_*m*1_ ^2^, *σ*_*m*2_ ^2^	20–69	2.02 (1.6–2.6)	1.29 (0.3–6.4)	1.68 (1.5–5.5)	−3950.978
					
*σ*_*p*_ ^2^(*t*)=*σ*_*m*_ ^2^(*t*)	20–29	3.50 (0.8–15.8)	3.50 (0.8–15.8)	3.50 (0.8–15.8)	−3948.721
	30–39	1.64 (0.8–3.6)	1.64 (0.8–3.6)	1.64 (0.8–3.6)	
	40–49	2.80 (1.8–4.5)	2.80 (1.8–4.5)	2.80 (1.8–4.5)	
	50–59	1.83 (1.0–3.4)	1.83 (1.0–3.4)	1.83 (1.0–3.4)	
	60–69	0.72 (0.2–2.6)	0.72 (0.2–2.6)	0.72 (0.2–2.6)	
					
*σ*_*p*_ ^2^(*t*), *σ*_*m*_ ^2^	20–29	3.59 (0.5–25.9)	1.59 (0.6–4.4)	1.59 (0.6–4.4)	−3949.089
	30–39	2.20 (1.0–4.9)	1.59 (0.6–4.4)	1.59 (0.6–4.4)	
	40–49	2.71 (1.7–4.3)	1.59 (0.6–4.4)	1.59 (0.6–4.4)	
	50–59	1.61 (0.8–3.1)	1.59 (0.6–4.4)	1.59 (0.6–4.4)	
	60–69	0.86 (0.2–3.2)	1.59 (0.6–4.4)	1.59 (0.6–4.4)	
					
*σ*_*p*_ ^2^(*t*), *σ*_*m*1_ ^2^, *σ*_*m*2_ ^2^	20–29	3.64 (0.5–26.9)	1.32 (0.3–6.2)	1.73 (0.6–5.4)	−3949.043
	30–39	2.19 (1.0–4.9)	1.32 (0.3–6.2)	1.73 (0.6–5.4)	
	40–49	2.71 (1.7–4.3)	1.32 (0.3–6.2)	1.73 (0.6–5.4)	
	50–59	1.61 (0.8–3.1)	1.32 (0.3–6.2)	1.73 (0.6–5.4)	
	60–69	0.87 (0.2–3.2)	1.32 (0.3–6.2)	1.73 (0.6–5.4)	

*σ_p_* ^2^(*t*)=polygenic variance; *σ_m_* ^2^(*t*)=modifying variance; *σ*_*m*1_ ^2^=*BRCA1* modifying variance; *σ*_*m*2_ ^2^=*BRCA2* modifying variance; Log-lik=log likelihood.

**Table 4:** Estimated *BRCA1* and *BRCA2* allele frequencies and relative hazards for model *σ_p_* ^2^=*σ_m_* ^2^ in Table 3

	***BRCA1***			***BRCA2***		
**Age (years)**	**Frequency (95% Cl)**	**BC RH (95% Cl)**	**OC RH (95% Cl)**	**Frequency (95% Cl)**	**BC RH (95% Cl)**	**OC RH (95% Cl)**
	0.0006 (0.04–0.10%)			0.0010 (0.06–0.15%)		
20–29		44.9 (25.4–79.2)	1.0		21.1 (10.9–40.6)	1.0
30–39		26.2 (18.3–37.3)	39.0 (17.2–88.5)		14.8 (10.2–21.4)	1.0
40–49		19.9 (14.3–27.7)	68.4 (43.5–107.5)		11.0 (7.8–15.5)	3.9 (0.8–19.4)
50–59		9.6 (5.8–15.9)	26.2 (13.6–50.2)		9.7 (6.3–14.7)	19.4 (10.4–36.3)
60–69		8.3 (4.0–17.3)	37.8 (17.8–80.2)		9.1 (5.1–16.1)	7.1 (1.8–28.3)

BC=breast cancer; OC=ovarian cancer; RH=relative hazard.

RH are estimated relative to the cohort-specific population incidences. RH assumed to be constant across birth cohorts.

**Table 5:** Age-specific observed (Collaborative Group in Hormonal Factors in Breast Cancer, 2001) and predicted FRRs of breast cancer associated with having an affected mother

	**Predicted FRR**			
**Age (years)**	***σ_p_*^2^=*σ_m_*^2a^**	***σ_p_*^2^(*t*)=*σ_m_*^2^(*t*)=*α*+*βt***	***σ_p_*^2^(*t*)=*σ_m_*^2^(*t*)=*α*+*βt*** **b**	**Observed FRR (95% Cl)**
25	5.3	6.7	6.0	
30	3.1	3.9	5.8	5.7 (2.7–11.8)
35	2.9	3.4	3.5	
40	2.4	2.6	2.6	
45	2.2	2.3	2.2	2.0 (1.5–2.8)
50	2.0	1.9	1.9	
55	1.9	1.7	1.7	1.6 (1.2–2.1)
60	1.8	1.5	1.5	
65	1.7	1.4	1.4	1.4 (1.2–1.7)
70	1.7	1.3	1.3	

FRR=familial relative risk.

a These models refer to the models in Table 3. *BRCA1*/*2* log-relative hazards assumed to be constant in each decade of age.

b *BRCA1*/*2* log-relative hazards assumed to be piecewise linear functions of age.

**Table 6:** Parameter estimates for the final model

**Parameter**	***BRCA1***		***BRCA2***		**Noncarriers**	
Allele frequency (95% Cl)	0.0006 (0.04–0.10%)		0.0010 (0.07–0.16%)		NA	
						
Average penetrance (%) by the age of 70 years, by the year of birth						
						
	**Breast cancer**	**Ovarian cancer**	**Breast cancer**	**Ovarian cancer**	**Breast cancer**	**Ovarian cancer**
<1920	46	33	39	11	4.5	1.0
1920–1929	51	36	44	11	5.2	1.1
1930–1939	55	36	47	12	5.9	1.1
1940–1949	58	34	50	11	6.4	1.1
1950+	59	34	51	11	6.5	1.1
						
Polygenic modifying variance: *σ*_*p*_ ^2^(*t*)=*σ*_*m*_ ^2^(*t*)=*α*+*βt*						
	*α̂*=4.83 (95% Cl: 1.8–7.9)			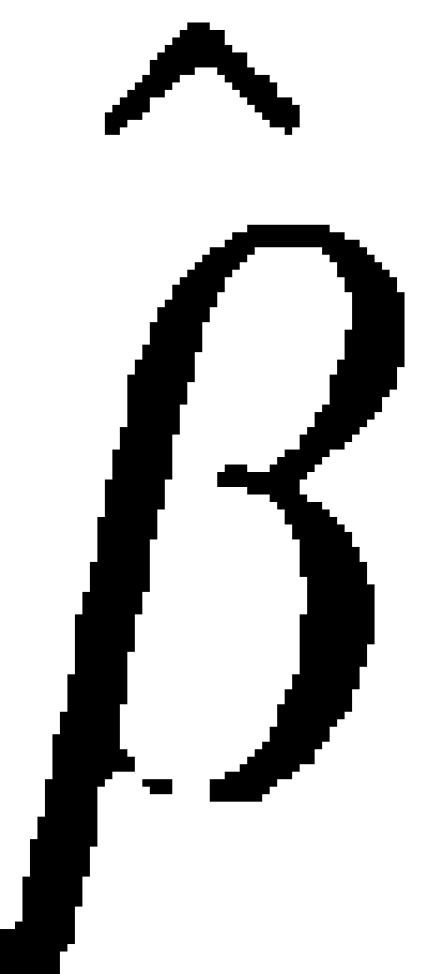 =−0.06 (95% Cl: −0.12 to 0.00)		
						

NA=not available.

Polygenic/modifying variance is a linear function of age, and the log *BRCA1* and *BRCA2* relative hazards are piecewise linear functions of age.

**Table 7:** Assumed relative risk parameters for the cancer risks at sites other than breast and ovary

		**Relative risk**	
**Site**	**Age group (years)**	***BRCA1***	***BRCA2***
Male breast		8.00^a^	80.00^b^
Prostate	<65	1.82^c^	7.33^d^
	⩾65	0.84^c^	3.39^d^
Pancreatic	<65	3.10^c^	5.54^d^
	⩾65	1.54^c^	1.61^d^

a DF Easton (unpublished data).

b Thompson and Easton (2001).

c Thompson and Easton (2002).

d The Breast Cancer Linkage Consortium (1999).

## APPENDIX

To obtain *BRCA1* and *BRCA2* incidences as continuous functions of age, we fitted models in which the log-relative hazards (*G*(*t*)) were piecewise linear functions of age. For example,
