# The BOADICEA model of genetic susceptibility to breast and ovarian cancers: updates and extensions

**DOI:** 10.1038/sj.bjc.6604411

**Published:** 2008-06-10

**Authors:** A C Antoniou, A P Cunningham, J Peto, D G Evans, F Lalloo, S A Narod, H A Risch, J E Eyfjord, J L Hopper, M C Southey, H Olsson, O Johannsson, A Borg, B Pasini, P Radice, S Manoukian, D M Eccles, N Tang, E Olah, H Anton-Culver, E Warner, J Lubinski, J Gronwald, B Gorski, L Tryggvadottir, K Syrjakoski, O-P Kallioniemi, H Eerola, H Nevanlinna, P D P Pharoah, D F Easton

**Correction to:** *British Journal of Cancer* (2008) **98**, 1457–1466; doi:10.1038/sj.bjc.6604305

Owing to an error on the part of the authors, co-author Barbara Pasini's surname was incorrectly submitted to the journal as ‘Passini’ and subsequently published as such. The correct spelling along with the complete list of authors is given above.

